# Exact traveling wave solutions of the KP-BBM equation by using the new approach of generalized (*G*′/*G*)-expansion method

**DOI:** 10.1186/2193-1801-2-617

**Published:** 2013-11-19

**Authors:** Md Nur Alam, M Ali Akbar

**Affiliations:** Department of Mathematics, Pabna University of Science and Technology, Pabna, Bangladesh; Department of Applied Mathematics, University of Rajshahi, Rahjshahi, Bangladesh

**Keywords:** New generalized (*G*′/*G*)-expansion method, KP-BBM equation, Exact solutions, Nonlinear partial differential equation, Homogeneous balance, Traveling wave solutions, Solitary wave solutions, Mathematics subject classification, 35C07, 35C08, 35P99

## Abstract

The new approach of the generalized (*G*′/*G*)-expansion method is an effective and powerful mathematical tool in finding exact traveling wave solutions of nonlinear evolution equations (NLEEs) in science, engineering and mathematical physics. In this article, the new approach of the generalized (*G*′/*G*)-expansion method is applied to construct traveling wave solutions of the Kadomtsev-Petviashvili-Benjamin-Bona-Mahony (KP-BBM) equation. The solutions are expressed in terms of the hyperbolic functions, the trigonometric functions and the rational functions. By means of this scheme, we found some new traveling wave solutions of the above mentioned equation.

## Introduction

The world around us is inherently nonlinear and NLEEs are widely used as models to describe the complex physical phenomena. The exact traveling wave solutions of NLEEs play a vital role in nonlinear science and engineering. Therefore, investigating traveling wave solutions is becoming increasingly attractive in nonlinear sciences day by day. However, not all equations posed of these models are solvable. As a result, many new techniques have been successfully developed by diverse group of scientists, such as, the rank analysis method (Feng 2000), the complex hyperbolic function method (Zayed et al. 2006; Chow 1995), the generalized Riccati equation method (Yan and Zhang 2001; Porubov 1996), the Jacobi elliptic function method (Chen and Wang 2005; Xu 2006; Yusufoglu and Bekir 2008; Zayed et al. 2004a), the ansatz method (Hu [Bibr CR20][Bibr CR21]), the Adomian decomposition method (Wazwaz 2002), the He’s homotopy perturbation method (Ganji and Rafei 2006; Ganji 2006; Ganji et al. 2007), the homogeneous balance method (Wang 1995,1996; Zayed et al. 2004b), the inverse scattering transform method (Ablowitz and Clarkson 1991), the Darboux transformation method (Matveev and Salle 1991), the Backlund transformation method (Miura 1978), the (*G*′/*G*)-expansion method (Wang et al. 2008; Akbar et al. 2012a, 2012b, 2012c, 2012d, 2012e; Bekir 2008; Zayed 2009; Zhang et al. 2008), the improved (*G*′/*G*)-expansion method (Zhang et al. 2010), the modified simple equation method (Jawad et al. 2010; Khan et al. 2013; Zayed and Ibrahim 2012), the Exp-function method (He and Wu 2006; Akbar and Ali 2012; Mohyud-Din et al. 2010), the tanh-function method (Malfliet 1992; Fan 2000), the sine-cosine method (Wazwaz 2004,2005; Bibi and Mohyud-Din 2013), the first integral method (Feng 2002; Tascan and Bekir 2010) etc.

Recently, the new generalized (*G*′/*G*) expansion method has been initiated by Naher and Abdullah (2013). The significance of the new generalized (*G*′/*G*) expansion method is that one can treat the nonlinear problems by essentially linear method. Moreover, it transforms a nonlinear evolution equation to a simple algebraic computation. The merits of the new generalized (*G*′/*G*) expansion method over the other methods are that it gives more general solutions with some free parameters and it handles NLEEs in a direct manner with no requirement for initial/boundary conditions or initial trial function at the outset.

Our aim in this paper is to present an application of the new generalized (*G*′/*G*) expansion method to solve the KP-BBM equation by using this method for the first time.

The rest of the paper is organized as follows: In Section “Description of the new generalized (G′/G)-expansion method”, we give the description of the method. In Section “Application of the method”, we exert this method to the KP-BBM equation. In Section “Discussions”, Discussions are presented. Conclusions are given in Section “Conclusion”.

## Description of the new generalized (*G*′/*G*)-expansion method

Let us consider a general nonlinear PDE in the form1

where *u* = *u*(*x*, *t*) is an unknown function, *P* is a polynomial in *u*(*x*, *t*) and its derivatives wherein the highest order derivatives and nonlinear terms are involved and the subscripts are used for the partial derivatives.

### Step 1

We combine the real variables *x* and *t* by a compound variable *ξ*:2

where *V* is the speed of the traveling wave. The traveling wave transformation (2) converts Eq. (1) into an ordinary differential equation (ODE) for *u* = *u*(*ξ*):3

where *Q* is a polynomial of u and its derivatives and the superscripts indicate the ordinary derivatives with respect to *ξ*.

### Step 2

According to possibility Eq. (3) can be integrated term by term one or more times, yields constant(s) of integration. The integral constant may be zero, for simplicity.

### Step 3

Suppose the traveling wave solution of Eq. (3) can be expressed as follows:4

where either *a*_*N*_ or *b*_*N*_ may be zero, but both *a*_*N*_ and *b*_*N*_ could be zero at a time, *a*_*i*_ (*i* = 0, 1, 2,…, *N*) and *b*_*i*_ (*i* = 1, 2,…, *N*) and *d* are arbitrary constants to be determined later and *H*(*ξ*) is5

where *G* = *G*(*ξ*) satisfies the following auxiliary equation:6

where the prime stands for derivative with respect to *ξ*; *A*, *B*, *C* and *E* are real parameters.

### Step 4

To determine the positive integer *N*, taking the homogeneous balance between the highest order nonlinear terms and the derivatives of the highest order appearing in Eq. (3).

### Step 5

Substitute Eq. (4) and Eq. (6) including Eq. (5) into Eq. (3) with the value of *N* obtained in Step 4, we obtain polynomials in (*d* + *H*)^*N*^ (*N* = 0, 1, 2,…) and (*d* + *H*)^−*N*^ (*N* = 0, 1, 2,…). We collect each coefficient of the resulted polynomials and setting them to zero yields a set of algebraic equations for *a*_*i*_ (*i* = 0, 1, 2,…, *N*) and *b*_*i*_ (*i* = 1, 2,…, *N*), *d* and *V*.

### Step 6

Suppose that the value of the constants *a*_*i*_ (*i* = 0, 1, 2,…, *N*),*b*_*i*_ (*i* = 1, 2,…, *N*), *d* and *V* can be found by solving the algebraic equations obtained in Step 5. Since the general solution of Eq. (6) is well known to us, inserting the values of *a*_*i*_ (*i* = 0, 1, 2,…, *N*), *b*_*i*_ (*i* = 1, 2,…, *N*), *d* and *V* into Eq. (4), we obtain more general type and new exact traveling wave solutions of the nonlinear partial differential equation (1).

Using the general solution of Eq. (6), we have the following solutions of Eq. (5):

### Family 1

When *B* ≠ 0, *Ψ* = *A* − *C* and Ω = *B*^2^ + 4*E*(*A* − *C*) > 0,7

### Family 2

When *B* ≠ 0, *Ψ* = *A* − *C* and Ω = *B*^2^ + 4*E*(*A* − *C*) < 0,8

### Family 3

When *B* ≠ 0, *Ψ* = *A* − *C* and Ω = *B*^2^ + 4*E*(*A* − *C*) = 0,9

### Family 4

When *B* = 0, *Ψ* = *A* − *C* and ∆ = *ΨE* > 0,10

### Family 5

When *B* = 0, *Ψ* = *A* − *C* and ∆ = *ΨE* < 0,11

## Application of the method

In this section, we will bring to bear the new generalized (*G*′/*G*) expansion method to construct new and more general traveling wave solutions of the KP-BBM equation. Let us consider the KP-BBM equation12

Now, we use the wave transformation (2) into the Eq. (12), which yields13

Integrating Eq. (13) twice with respect to *ξ*, we obtain14

where *P* is an integral constant which is to be determined.

Taking the homogeneous balance between *u*^2^ and *u*″ in Eq. (14), we obtain *N* = 2. Therefore, the solution of Eq. (14) is of the form:15

where *a*_0_, *a*_1_, *a*_2_, *b*_1_, *b*_2_ and *d* are constants to be determined.

Substituting Eq. (15) together with Eqs. (5) and (6) into Eq. (14), the left-hand side is converted into polynomials in (*d* + *H*)^*N*^(*N* = 0, 1, 2, ……) and (*d* + *H*)^−*N*^(*N* = 1, 2,…). We collect each coefficient of these resulted polynomials and setting them zero yields a set of simultaneous algebraic equations (for simplicity the equations are not presented here) for *a*_0_, *a*_1_, *a*_2_, *b*_1_, *b*_2_*d*, *P* and *V*. Solving these algebraic equations with the help of symbolic computation software Maple, we obtain following:

### Set 1

*P* = 0, *V* = *V*, *d* = *d*, *a*_1_ = 0, *a*_2_ = 0,16

where *Ψ* = *A* − *C*, *V*, *d*, *A*, *B*, *C*, *E* are free parameters.

### Set 2

*P* = 0, *V* = *V*, *d* = *d*, *b*_1_ = 0, *b*_2_ = 0,17

where *Ψ* = *A* − *C*, *V*, *d*, *A*, *B*, *C*, *E* are free parameters.

### Set 3

*P* = 0, *V* = *V*, *a*_1_ = 0, *b*_1_ = 0,18

where *Ψ* = *A* − *C*, *V*, *A*, *B*, *C*, *E* are free parameters.

For set 1, substituting Eq. (16) into Eq. (15), along with Eq. (7) and simplifying, yields following traveling wave solutions (if *C*_1_ = 0 but *C*_2_ ≠ 0; *C*_2_ = 0 but *C*_1_ ≠ 0) respectively:

Substituting Eq. (16) into Eq. (15), along with Eq. (8) and simplifying, the exact solutions become (if *C*_1_ = 0 but *C*_2_ ≠ 0; *C*_2_ = 0 but *C*_1_ ≠ 0):

Substituting Eq. (16) into Eq. (15), together with Eq. (9) and simplifying, the obtained solution becomes:

Substituting Eq. (16) into Eq. (15), along with Eq. (10) and simplifying, we obtain following traveling wave solutions (if *C*_1_ = 0 but *C*_2_ ≠ 0; *C*_2_ = 0 but *C*_1_ ≠ 0):

Substituting Eq. (16) into Eq. (15), together with Eq. (11) and simplifying, the obtained exact solutions become (if *C*_1_ = 0 but *C*_2_ ≠ 0; *C*_2_ = 0 but *C*_1_ ≠ 0) respectively:

where *ξ* = *x* − *Vt*.

Similarly, for set 2, substituting Eq. (17) into Eq. (15), along with Eq. (7) and simplifying, the traveling wave solutions become (if *C*_1_ = 0 but *C*_2_ ≠ 0; *C*_2_ = 0 but *C*_1_ ≠ 0) respectively:

Substituting Eq. (17) into Eq. (15), along with Eq. (8) and simplifying, yields exact solutions (if *C*_1_ = 0 but *C*_2_ ≠ 0; *C*_2_ = 0 but *C*_1_ ≠ 0) respectively:

Substituting Eq. (17) into Eq. (15), along with Eq. (9) and simplifying, our obtained solution becomes:

Substituting Eq. (17) into Eq. (15), together with Eq. (10) and simplifying, yields following traveling wave solutions (if *C*_1_ = 0 but *C*_2_ ≠ 0; *C*_2_ = 0 but *C*_1_ ≠ 0) respectively:

Substituting Eq. (17) into Eq. (15), along with Eq. (11) and simplifying, our exact solutions become (if *C*_1_ = 0 but *C*_2_ ≠ 0; *C*_2_ = 0 but *C*_1_ ≠ 0) respectively:

where *ξ* = *x* − *Vt*.

Similarly, For set 3, substituting Eq. (18) into Eq. (15), together with Eq. (7) and simplifying, yields following traveling wave solutions (if *C*_1_ = 0 but *C*_2_ ≠ 0; *C*_2_ = 0 but *C*_1_ ≠ 0) respectively:

Substituting Eq. (18) into Eq. (15), along with Eq. (8) and simplifying, we obtain following solutions (if *C*_1_ = 0 but *C*_2_ ≠ 0; *C*_2_ = 0 but *C*_1_ ≠ 0) respectively:

Substituting Eq. (18) into Eq. (15), along with Eq. (9) and simplifying, our obtained solution becomes:

Substituting Eq. (18) into Eq. (15), along with Eq. (10) and simplifying, yields following exact traveling wave solutions (if *C*_1_ = 0 but *C*_2_ ≠ 0; *C*_2_ = 0 but *C*_1_ ≠ 0) respectively:

Substituting Eq. (18) into Eq. (15), along with Eq. (11) and simplifying, the obtained solutions become (if *C*_1_ = 0 but *C*_2_ ≠ 0; *C*_2_ = 0 but *C*_1_ ≠ 0) respectively:

where *ξ* = *x* − *Vt*.

### Remark

The solutions obtained in this article have been checked by putting them back into the original equation and found correct.

## Discussions

The advantages and validity of the method over the basic (*G*′/*G*)-expansion method have been discussed in the following.

### Advantages

The crucial advantage of the new generalized (*G*′/*G*)-expansion method over the basic (*G*′/*G*)-expansion method is that the method provides more general and large amount of new exact traveling wave solutions with several free parameters. The exact solutions have its great importance to expose the inner mechanism of the complex physical phenomena. Apart from the physical application, the close-form solutions of nonlinear evolution equations assist the numerical solvers to compare the accuracy of their results and help them in the stability analysis.

### Validity

Feng and Zheng (2010) investigated the well-established KP-BBM equation to obtain exact solutions via the basic (*G*′/*G*)-expansion method and achieved only three solutions (A.1),(A.2),(A.3) (see Appendix). On the other hand, twenty seven solutions are constructed of this equation by applying the new approach of generalized (*G*′/*G*)-expansion method. They used the linear ordinary differential equation as an auxiliary equation and traveling wave solutions presented in the form  where *a*_*m*_ ≠ 0. It is noteworthy to point out that some of our solutions are coincided with the solutions obtained by Feng and Zheng (2010) if the parameters are taken particular values, which validate our solutions.

## Conclusion

The new generalized (*G*′/*G*)-expansion method established by Naher and Abdullah has successfully been implemented to construct new and more general exact traveling wave solutions of the KP-BBM equation. The method offers solutions with free parameters that might be important to explain some complex physical phenomena. Comparing the currently proposed method with other methods, such as (*G*′/*G*)-expansion method, the Exp-function method and the modified simple equation method, we might conclude that the exact solutions to Eq. (12) can be investigated by simple and systematic way. This study shows that the new generalized (*G*′/*G*)-expansion method is quite efficient and practically well suited to be used in finding exact solutions of NLEEs. Also, we observe that the new generalized (*G*′/*G*)-expansion method is straightforward and can be applied to many other nonlinear evolution equations.

## Appendix

### Feng and Zheng’s solutions (Feng and Zheng 2010)

Feng and Zheng (2010) established exact solutions of the well-known the KP-BBM equation by using the basic (*G*′/*G*)-expansion method which are as follows:

When *λ*^2^ − 4 *μ* > 0,A.1

where  and *C*_1_, *C*_2_ are arbitrary constants.

When *λ*^2^ − 4 *μ* < 0,A.2

where  and *C*_1_, *C*_2_ are arbitrary constants.

When *λ*^2^ − 4 *μ* = 0,A.3

where  and *C*_1_, *C*_2_ are arbitrary constants.
